# The Great Escape: Pathogen Versus Host

**DOI:** 10.1371/journal.ppat.1004661

**Published:** 2015-03-12

**Authors:** Eric Kong, Mary Ann Jabra-Rizk

**Affiliations:** 1 Graduate Program in Life Sciences, Molecular Microbiology and Immunology Program, University of Maryland, Baltimore, Maryland, United States of America; 2 Department of Oncology and Diagnostic Sciences, Dental School, University of Maryland, Baltimore, Maryland, United States of America; 3 Department of Microbiology and Immunology, School of Medicine, University of Maryland, Baltimore, Maryland, United States of America; McGill University, CANADA

## Introduction

When bodily surface barriers have been breached, invading microorganisms are confronted by the innate immune system [1]. The first step in mounting a protective response is the rapid activation of an acute inflammatory response characterized by the migration and accumulation of immune cells at the site of invasion. As a primary defense against microbial infections, professional phagocytic cells such as macrophages will attempt to engulf and dispose of the invading microorganisms and their products. The recognition by effector cells of the innate immune system initiates signaling cascades, resulting in phagocytosis, secretion of microbicidal compounds and production of proinflammatory mediators. These early events culminate in the activation of adaptive immune responses; therefore, if launched early and effectively, innate immune responses limit the establishment of infectious foci and curb the severity of infections. However, it becomes more and more evident that microbial pathogens have developed very efficient strategies to circumvent and misguide host defenses, and therefore, their presence in the host results either in their elimination or in infection. Because of the critical role the innate immune system has in controlling microbial burden during the early stages of infection, the mechanisms employed by invading pathogens to thwart host immune defenses have attracted increasing interest. Here we synopsize some of the strategies exploited by two ubiquitous yet important human pathogens, the fungal species *Candida albicans* and the bacterial species *Staphylococcus aureus* [2,3]. In addition to possessing an array of virulence factors, these diverse species share many pathogenic characteristics, including the ability to form biofilms on host and abiotic surfaces, rapid development of antimicrobial resistance, and the ability to alter their transcriptome in response to stresses inflicted upon them by host immune cells. Importantly, although *C*. *albicans* and *S*. *aureus* are commensal species commonly colonizing various niches in the human host, they are the most frequent combination of organisms isolated from polymicrobial infections [4].

## *Staphylococcus aureus*: A Resourceful Bacterial Species

*Staphylococcus aureus* is a precarious microbial species carried by about 30% of the population and has been implicated in a variety of diseases, ranging from minor skin infections to serious invasive diseases [2]. When the outer physical barriers of the body, comprising skin and mucous surfaces, have been breached by *S*. *aureus*, the organism is confronted by the host's immune system, both innate and acquired. *S*. *aureus* infection of the skin stimulates a strong inflammatory response, involving the migration of neutrophils and macrophages to the site of infection. However, multiple strategies have made *S*. *aureus* exceptionally successful in subverting its human host, thereby promoting its spread [5]. Among the numerous immune evasion mechanisms deployed by *S*. *aureus* is secretion of proteins that inhibit opsonization, complement activation and chemotaxis, or that lyse neutrophils and neutralize antimicrobial peptides [5]. Specifically, staphyloccocal Protein A, present on the surface of *S*. *aureus*, can bind to the Fc region of host immunoglobulins, compromising the phagocytic ability of innate immune cells. In addition, *S*. *aureus* can inhibit the complement pathway by inhibiting the formation of the C1qrs complex in the classical pathway via collagen adhesion (Cna) or by degrading C3b via clumpling factor A (ClfA). Further, *S*. *aureus* is known to overproduce a subset of immunomodulatory proteins known as the staphylococcal superantigen-like proteins (Ssls), and a family of phenol-soluble modulins (PSMs) have emerged as novel toxins causing lysis of red and white blood cells [6]. Additionally, a cysteine protease, Staphopain A, was recently identified as a chemokine receptor blocker inhibiting neutrophil migration [7]. Similarly, a metalloprotease, aureolysin, was also shown to be a potent inhibitor of phagocytosis and killing of bacteria by neutrophils [8]. Importantly, *S*. *aureus* is capable of surviving in phagosomes of phagocytic cells by expressing superoxide dismutase enzymes that remove O_2_^-^ and release α-hemolysin to escape into the cytoplasm where it can remain viable in vacuolar compartments [9]. Later, through the expression of α-toxin, *S*. *aureus* can lyse the macrophage plasma membrane and escape into the surrounding environment [9]. Interestingly, findings from a recent study demonstrated that *S*. *aureus* is also capable of inducing immune cell death via secretion of a series of bacterial nucleases that degrade DNA released by neutrophils to trap immobilizing pathogens. Paradoxically, the degraded DNA components can ultimately activate caspase-3 in macrophages, thereby inducing apoptosis, which allows for staphylococcal persistence [10]. Not surprisingly, the ability of *S*. *aureus* to utilize such sophisticated systems has made it a model system for the study of novel virulence factors that compromise components of the innate immune system [5].

## *Candida albicans*: An Evolved Fungal Species

*Candida albicans* is the most common and major invasive fungal pathogen of humans, causing diseases ranging from superficial mucosal to disseminated, systemic infections that are often life-threatening [3]. As part of the commensal flora, *C*. *albicans* asymptomatically inhabits the mucosal surfaces of most healthy individuals. However, as an opportunistic pathogen, when host defenses are weakened, such as in AIDS, *C*. *albicans* can proliferate, causing serious infections [11]. As a complex and highly evolved pathogen, *C*. *albicans* has acquired efficient strategies to avoid contact with immune cells, and these strategies are often mediated by masking of immunostimulatory surface molecules [12]. Following recognition, phagocytes initiate engulfment of *C*. *albicans*, ultimately leading to the formation of a specialized organelle: the phagolysosome [12]. The microbicidal microenvironment of this organelle is associated with a reduction in pH, the presence of hydrolytic enzymes and antimicrobial peptides, and the generation of toxic oxidative compounds. Nevertheless, *C*. *albicans* has developed strategies to survive within phagocytes by suppressing the generation of toxic compounds via a secreted mediator compound [13]. The dramatic transcriptional and translational reprogramming exhibited by *C*. *albicans* inside the macrophage is indicative of its rapid induction of survival strategies and adaptation to the harsh internal environment of the phagocyte, which causes severe nutrient limitation, oxidative stress and phagosomal acidifcation [14].

The interaction of *C*. *albicans* with phagocytes is highly dynamic. As a dimorphic fungal species, *C*. *albicans* has the ability to respond to environmental factors and switch morphologies accordingly, between yeast and hyphal forms, a property central to its pathogenesis [15]. Therefore, the conditions in which *C*. *albicans* is growing, such as available nutrients, temperature and pH, are important in selectively favoring the yeast or hyphal form. Thus, although some of the strategies *C*. *albicans* uses to survive attack from phagocytes are also employed by bacteria, the role of morphology in escaping from phagocytes is unique to *C*. *albicans*. While phagocytic cells are able to kill *C*. *albicans*, most of the ingested yeast cells survive. However, phagocytosis induces a switch in *C*. *albicans* morphology from the yeast to the hyphal form where elongating hyphae puncture through the macrophage membrane. This results in lysis and killing of macrophages, thereby allowing *C*. *albicans* to escape [16]. Although it is not quite clear what triggers morphogenesis within the phagocyte where the environment is acidic, recent studies demonstrated that in addition to carbon dioxide produced by macrophages, exposure to sublethal reactive oxygen species (ROS) concentrations in the phagocyte up-regulates *C*. *albicans* arginine biosynthesis, allowing *C*. *albicans* to neutralize the phagosome, inducing germination and hyphal morphogenesis [17]. Therefore, in addition to pathogenesis, the yeast-to-hyphae transition plays a pivotal role in facilitating *C*. *albicans* escape from phagocytes and dissemination. However, macrophage death by ingested *C*. *albicans* is not only the result of physical rupture by hyphae, but, similar to what was reported for *S*. *aureus*, a new model was recently described where *C*. *albicans*–induced macrophage lysis also occurs via pyroptosis, a proinflammatory host cell–programmed death pathway independent of hyphal formation [16,18].

## *C*. *albicans*, *S*. *aureus*, and the Host

Polymicrobial infections caused by combinations of microorganisms are responsible for significant morbidity and mortality [19]. In these infections, the presence of one microorganism may predispose the host to colonization by others, and in additive polymicrobial infections, two or more nonpathogenic microorganisms together can cause disease [19]. *C*. *albicans* and *S*. *aureus* have been shown to co-adhere as they exist in mixed biofilms on host tissue and abiotic surfaces with *S*. *aureus* exhibiting high affinity to the *C*. *albicans* hyphae mediated by the hyphal-specific adhesin Als3p as a receptor [20]. Significantly, recent in vivo studies demonstrated a grave clinical implication to this interaction where using a mouse model, the co-colonization of *C*. *albicans* and *S*. *aureus* on oral tissue resulted in systemic staphylococcal infection despite the massive influx of host immune cells to site of infection [21]. Similar to the in vitro findings, in this model, Als3p was found to be crucial during the early stages of simultaneous co-colonization in the animals. In contrast, however, findings from a more recent study demonstrated that staphylococcal disease is not contingent upon Als3p once *C*. *albicans* colonization and infection are established [21,22]. Combined, these findings indicate that the process of *C*. *albicans*–*S*. *aureus* interaction is complex and multifactorial.

**Fig 1:**
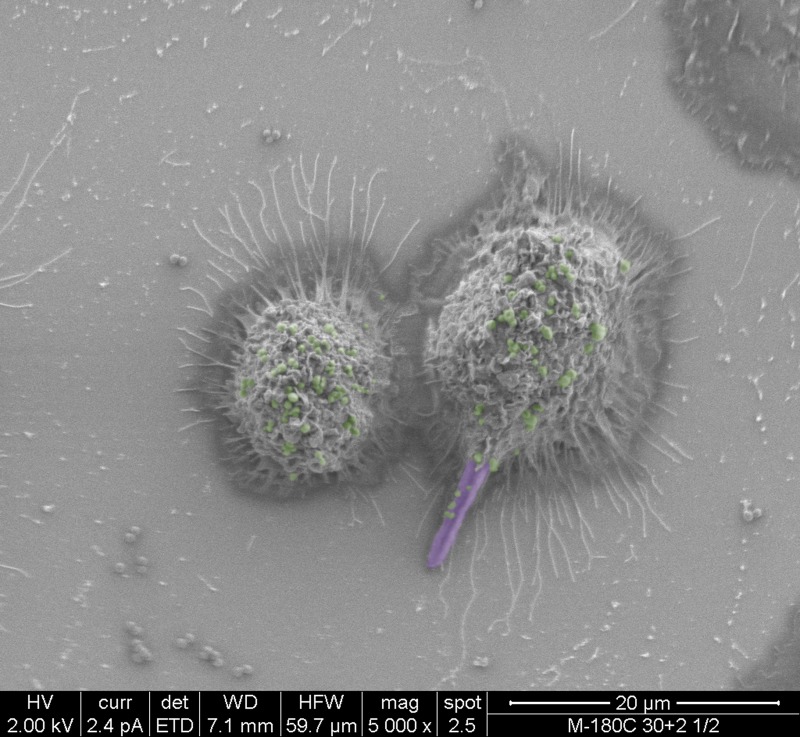
False-colored scanning electron micrographs depicting the co-phagocytosis of *C*. *albicans* and *S*. *aureus* by murine macrophages. Within one hour of co-ingestion, *C*. *albicans* hyphae formed inside the macrophage pierce the cell membrane with *S*. *aureus* seen adhering to the protruding hyphae. Ultimately, the macrophage lyses, releasing intracellular *S*. *aureus* cells. *C*. *albicans* hyphae are purple; *S*. *aureus* cells are green.

In addition to successful microbial persistence and enhanced virulence, infection by one pathogen can manipulate host immunity to the benefit of other pathogens. Using a mouse model of peritonitis, a recent study by Peters et al. [23] demonstrated that co-infection with *C*. *albicans* and *S*. *aureus* resulted in enhanced mortality, concomitant with significant increase in proinflammatory cytokines associated with an acute aggressive inflammatory response. Combined, the findings from these recent studies indicate that in addition to augmented pathogenicity, *C*. *albicans* and *S*. *aureus* co-infection modulate innate inflammatory events. Therefore, it is feasible to speculate that the interaction between these diverse species in a host may also involve immune co-evasion strategies to compromise the ability of immune cells against polymicrobial infections. These speculations were alluded to by co-phagocytosis studies using murine macrophages where *S*. *aureus* was seen co-escaping macrophages via its association with the invasive hyphae (Fig 1). Despite the important clinical implications, however, this aspect of fungal-bacterial interactions in the host is yet to be fully explored.

## Future Perspectives

Ample information is available on the various strategies employed by microbial species to resist and survive host immune defenses. However, there are significant gaps in our understanding of the mechanisms that regulate host innate immunity during simultaneous infection with multiple microbial species. As modulatory immunotherapies continue to be developed, a fuller understanding of the complex circuitry directing pathogen response to host defences may allow us to develop more effective treatments in the context of complex polymicrobial infections. Therefore, future studies should be directed towards designing suitable animal models to explore the impact of interspecies interactions on host immunity, particularly when they involve ubiquitous commensal species with high pathogenic potential.
